# Upregulation of Hox genes leading to caste-specific morphogenesis in a termite

**DOI:** 10.1186/s13227-023-00216-w

**Published:** 2023-07-27

**Authors:** Kohei Oguchi, Toru Miura

**Affiliations:** Misaki Marine Biological Station, Graduate School of Science, The University of Tokyo, Miura, Kanagawa 238-0225 Japan

**Keywords:** Termite, Caste differentiation, Hox genes, Post-embryonic development

## Abstract

**Background:**

In social insects, interactions among colony members trigger caste differentiation with morphological modifications. In termite caste differentiation, caste-specific morphologies (such as mandibles in soldiers, genital organs in reproductives or wings in alates) are well developed during post-embryonic development under endocrine controls (e.g., juvenile hormone and ecdysone). Since body part-specific morphogenesis in caste differentiation is hormonally regulated by global factors circulated throughout the body, positional information should be required for the caste-specific and also body part-specific morphogenesis. To identify factors providing the positional information, expression and functional analyses of eight Hox genes were carried out during the three types of caste differentiation (i.e., soldier, neotenic and alate differentiation) in a termite, *Hodotermopsis sjostedti*.

**Results:**

Spatio-temporal patterns of Hox gene expression during caste differentiation were elucidated by real-time qPCR, showing the caste-specific upregulations of Hox genes during the differentiation processes. Among eight Hox genes, *Deformed* (*Dfd*) was upregulated specifically in mandibles in soldier differentiation, *abdominal-A* (*abd-A*) and *Abdominal-B* (*Abd-B*) were upregulated in the abdomen in neotenic differentiation, while *Sex-comb reduced* (*Scr*) and *Antennapedia* (*Antp*) were upregulated during alate differentiation. Furthermore, RNAi knockdown of *Dfd* in soldier differentiation and of *abd-A* and *Abd-B* in neotenic differentiation distorted the modifications of caste-specific morphologies.

**Conclusions:**

Gene expression and functional analyses in this study revealed that, in the caste differentiation in termites, upregulation of Hox genes provide positional identities of body segments, resulting in the caste-specific morphogenesis. The acquisition of such developmental modifications would have enabled the evolution of sophisticated caste systems in termites.

**Supplementary Information:**

The online version contains supplementary material available at 10.1186/s13227-023-00216-w.

## Background

Animal body-plans are not only determined genetically, but are also altered by environmental stimuli during development, a phenomenon known as phenotypic plasticity and polyphenism [1]. Caste polyphenism in social insects, in which specific tasks are allocated to certain phenotypes (castes) to cooperatively perform social behaviors in a colony [1–3], is one of the representative examples of polyphenism. Caste-specific morphological characteristics specialized for allocated tasks develop during caste differentiation processes in response to extrinsic factors such as social interactions among colony members [4–6]. However, little is known about how these alternative phenotypes are derived from a single genome. Among the caste differentiations in various eusocial insects, the patterns of caste differentiation in termites (superfamily Termitoidea in order Blattodea) are distinctive, because in termites, castes can change through molting [5–9].

**Fig. 1:**
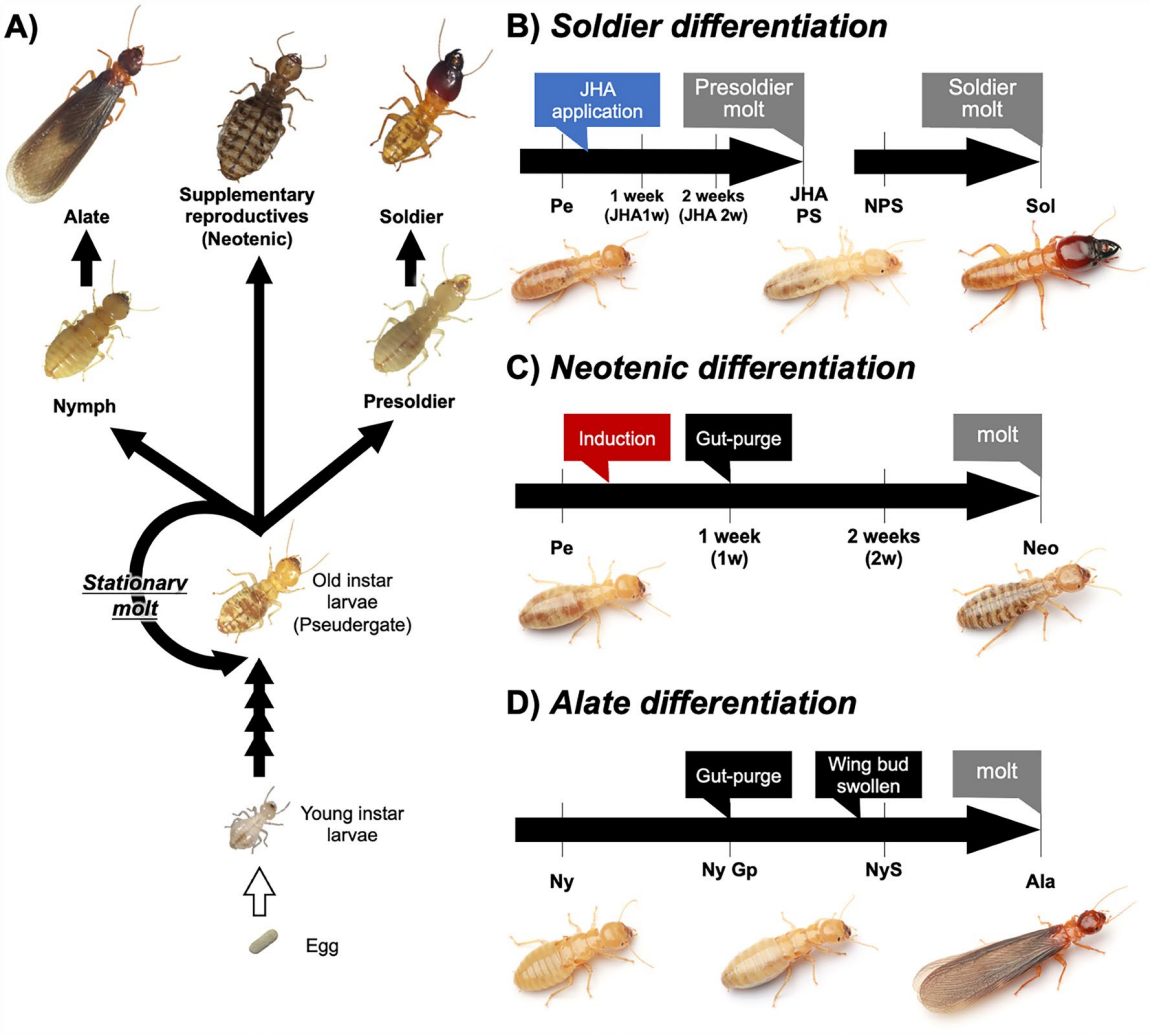
Caste differentiation pathway in *H. sjostedti* (**A**) and diagrams of sampling scheme used in this study (**B**–**D**). **B** Sampling schedules of soldier differentiation pathway, indicating six stages sampled for real-time qPCR (i.e., Pe, JHA1w, JHA2w, JHAPS, NPS and Sol). **C** Outline of the neotenic differentiation process, in which four developmental stages were compared by real-time qPCR (Pe, 1w, 2w, Neo). **D** In the process of alate differentiation, pseudergate and each nymphal stage (i.e., Pe, Ny, NyGp, NyS and Ala) were compared

In termites, caste differentiations occur from immature individuals, including pseudergates (i.e., false workers), in response to environmental stimuli such as individual interactions among colony members [10]. These immature individuals have the potential to differentiate into any caste, including alates, which molt via the nymph and found new colonies [11, 12]; soldiers, which differentiate via a presoldier stage and defend their colonies [7]; and neotenic reproductives, which differentiate from pseudergates when primary reproductives die or become senescent and take over the reproductive role [13]. During these caste differentiation processes, several body parts are specifically modified through caste-specific morphogenesis (Fig. 1) [5], such as wing development in alate differentiation [14, 15], mandibular or nasus modifications in soldier differentiation [16–19] and genital structure modifications in neotenic differentiation [20].

Caste differentiation in termites is regulated by endocrine factors such as juvenile hormone (JH) and ecdysone [21–23]. In many termite species, the application of JH or its analogue (JHA) soldier differenatiation accompanied by morphological changes [24]. Knockdown of the JH receptor gene *Methoprene-tolerant* (*Met*) and *ecdysone receptor* (*EcR)* was shown to inhibit soldier differentiation [25, 26] and knockdown of genes downstream of ecdysone, such as ecdysone-induced transcription factor *E93*, disrupted genital structure modifications [27], indicating that the downstream genes of JH and ecdysone play important roles, respectively, in the soldier- and neotenic-specific morphogenesis [6, 23, 28]. Since endocrine factors are global factors, which can circulate throughout a body, it is predicted that there should be some interplays between such global hormonal factors and morphogenetic factors providing positional information of body parts, leading to the body parts-specific morphogenesis. Although previous studies have demonstrated such physiological links [19], it is not clear how various forms of caste phenotypes emerge from the same genetic information.

To elucidate the mechanisms underlying body part-specific morphogenesis during caste differentiation, we focused on the expression patterns and functions of morphogenetic regulatory genes, i.e., *Hox* genes that determine the identity of body parts along the anterior–posterior axis and are widely conserved among animals [29–31]. In this study, using *Hodotermopsis sjostedti* in which artificial induction methods of soldier and neotenic differentiation were established [32, 33], the expression patterns of eight *Hox* genes (*labial* [*lab*], *proboscipedia* [*pb*], *Deformed* [*Dfd*], *Sex combs reduced* [*Scr*], *Antennapedia* [*Antp*], *Ultrabithorax* [*Ubx*], *abdominal-A* [*abd-A*], and *Abdominal-B* [*Abd-B*]) were investigated. The temporal and spatial expression patterns were analyzed during several developmental stages (Fig. 1B–D) and in three body parts (i.e., head, thorax, abdomen). The results suggest that different Hox genes are specifically upregulated depending on the caste fates, contributing to the body parts and caste-specific morphogenesis.

## Results

### Expression profiles of Hox genes during caste differentiation

**Fig. 2:**
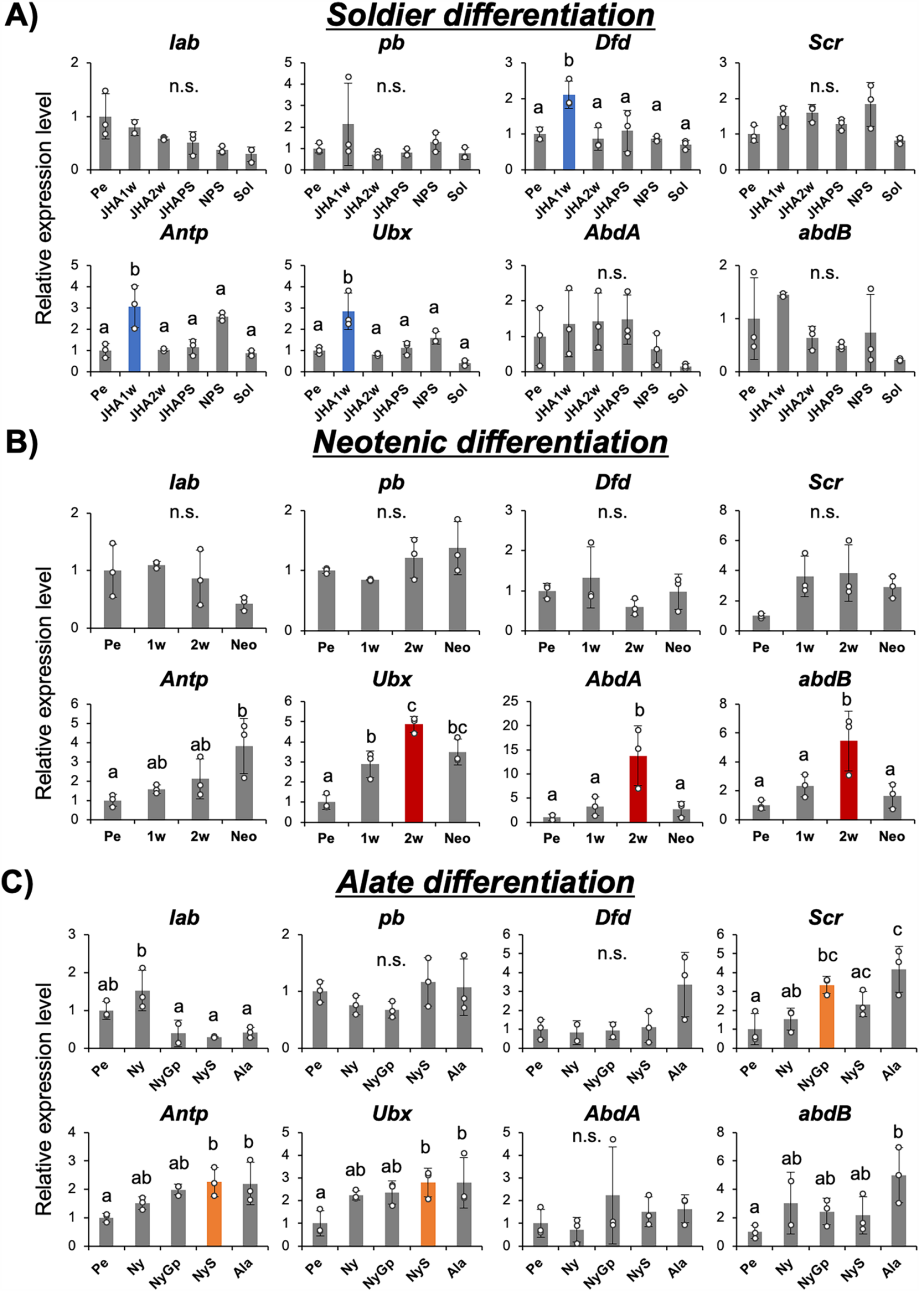
Gene expression profiles of eight Hox genes during soldier and neotenic differentiation. Expression levels (mean ± S.D., *n* = 3) relative to the mean expression in pseudergates were calibrated using the selected internal control (RP49). The dot plot indicates actual expression levels in each sample. Different letters above the bars indicate significant differences between groups (Tukey’s test, *p* < 0.05). **A** Among eight genes, *Dfd, Antp* and *Ubx* were highly expressed 1 week after induction of soldier differentiation, highlighted by blue bars. **B** Before neotenic differentiation, *Ubx, abdA* and *AbdB* were highly expressed, indicated by red bars. **C** During alate differentiation, *Scr*, *Antp* and *Ubx* were upregulated

To specify the genes that were highly expressed before the differentiation into soldiers, neotenics and alates, the expression levels of eight *Hox* genes were firstly compared among caste differentiation pathways (Fig. 1B–C). Among these eight *Hox* genes, *Dfd, Antp* and *Ubx* were more highly expressed in pseudergates 1 week after the induction of soldier differentiation, while *lab*, *pb, Scr, abd-A* and *Abd-B* were not highly expressed before the presoldier molt (Fig. 2A, Tukey’s test; *p* < 0.05). By contrast, during neotenic differentiation, *Ubx, abd-A* and *Abd-B* were more highly expressed at around 2 weeks in pseudergates, while the other genes were not highly expressed before the molt (Fig. 2B, Tukey’s test; *p* < 0.05). In addition, *Scr*, *Antp* and *Ubx* were upregulated in nymphs before the alate molt, while other genes showed no significant expression changes before this molt (Fig. 2C, Tukey’s test; *p* < 0.05). Therefore, for each type of caste differentiation, three sets of three different Hox genes [*Dfd, Antp* and *Ubx* for soldier differentiation], [*Ubx, abd-A* and *Abd-B* for neotenic differentiation] and [*Scr*, *Antp* and *Ubx* for alate differentiation] were upregulated.

### Expression profiles of Hox genes among body parts

**Fig. 3:**
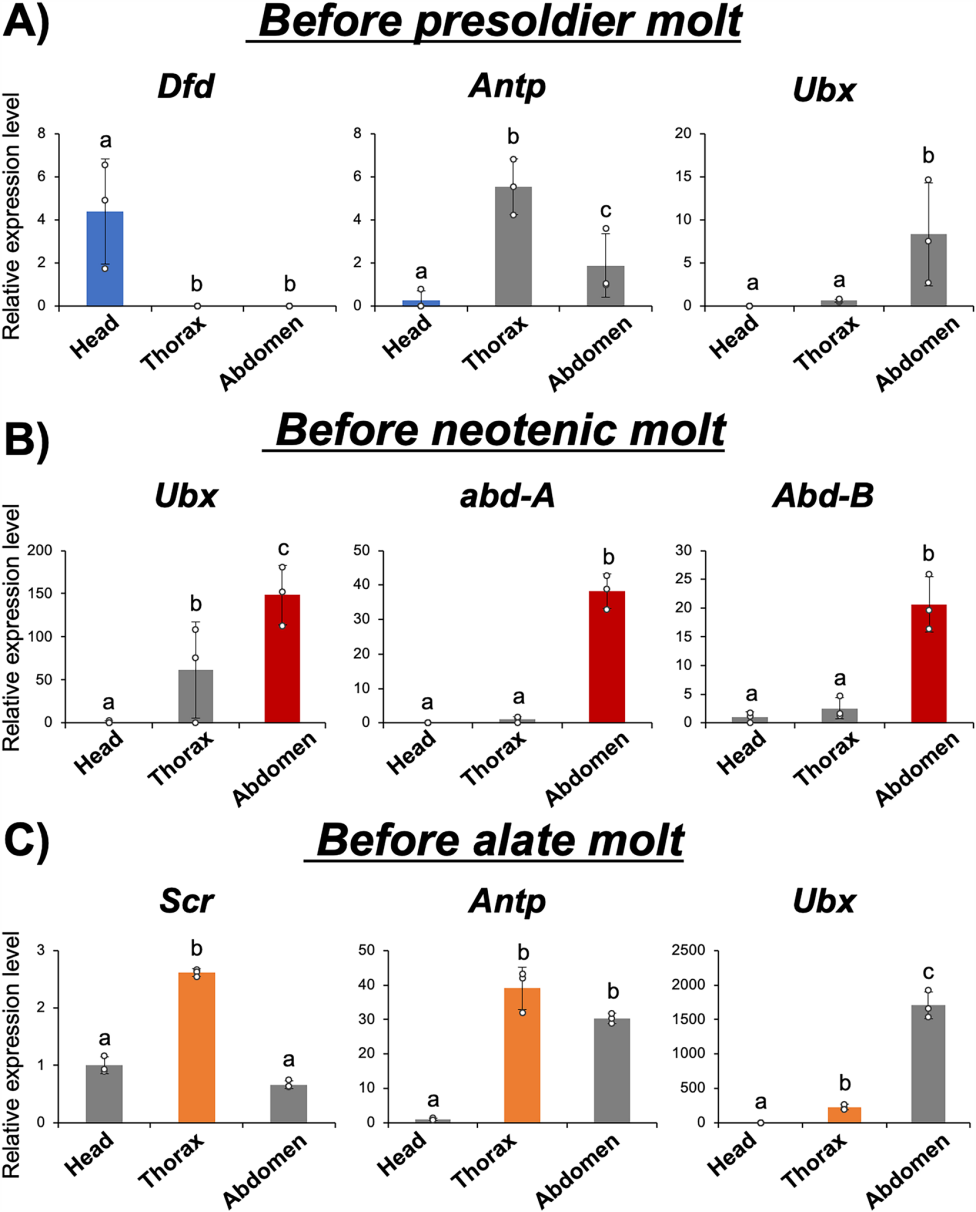
Gene expression profiles of highly expressed genes before presoldier and neotenic molt were compared among three body parts (i.e., head, thorax and abdomen). Relative expression levels (mean ± S.D., *n* = 3) to the mean expression were calibrated using an internal control (RP49). The dots on each bar indicate actual expression levels in each sample. **A** At 1 week after induction of presoldier molt, only *Dfd* was highly expressed at the head (highlighted in blue). **B** 2 weeks after induction of neotenic differentiation, *abd-A* and *Abd-B* were specifically expressed at the abdomen (highlighted in red). **C** In nymphs with swollen wing buds, at stages just prior to alate molt, *Scr* and *Antp* were specifically expressed at the thorax (highlighted in orange). Different letters shown above the bars indicate significant differences among categories (Tukey’s test, *p* < 0.05)

Since the above experiments showed the temporal dynamics of Hox gene expressions during caste differentiation (Fig. 2), the spatial patterns of Hox expressions were then analyzed by comparing among head, thorax and abdomen (Fig. 3). Gene expression analyses (qPCR) were carried out at the time when the focal Hox genes were highly upregulated. Namely, the expression levels of three genes (*Dfd, Antp* and *Ubx*) at 1 week after induction of soldier differentiation, those of three genes (*Ubx, abd-A* and *Abd-B*) at 2 weeks after induction of the neotenic molt, and those of three genes (*Scr, Antp* and *Ubx*) at the nymphal stage just prior to the alate molt (in nymphs with swollen wing buds) were compared among the head, thorax and abdomen (Fig. 3). During the soldier differentiation, only the expression of *Dfd* was significantly higher in the head before the presoldier molt (Fig. 3A, Tukey’s test; *p* < 0.05). During the neotenic differentiation, *abd-A* and *Abd-B* were shown to be expressed specifically in the abdomen. It was also found that *Ubx* was expressed highly in the abdomen compared to the head and thorax, but the difference was not as large as those for *abd-A* and *Abd-B* (Fig. 3B, Tukey’s test; *p* < 0.05). In the alate differentiation, *Scr* and *Antp* showed significantly higher expression in the thorax, while *Ubx* showed the highest expression in the abdomen (Fig. 3C). Therefore, *Dfd* in the head of soldier differentiation, *abd-A* and *Abd-B* in the abdomen of neotenic molt, and *Scr* and *Antp* in the thorax of the alate differentiation showed distinctive expression patterns in the respective caste differentiation.

### Functional analyses of Hox genes by RNAi

**Fig. 4:**
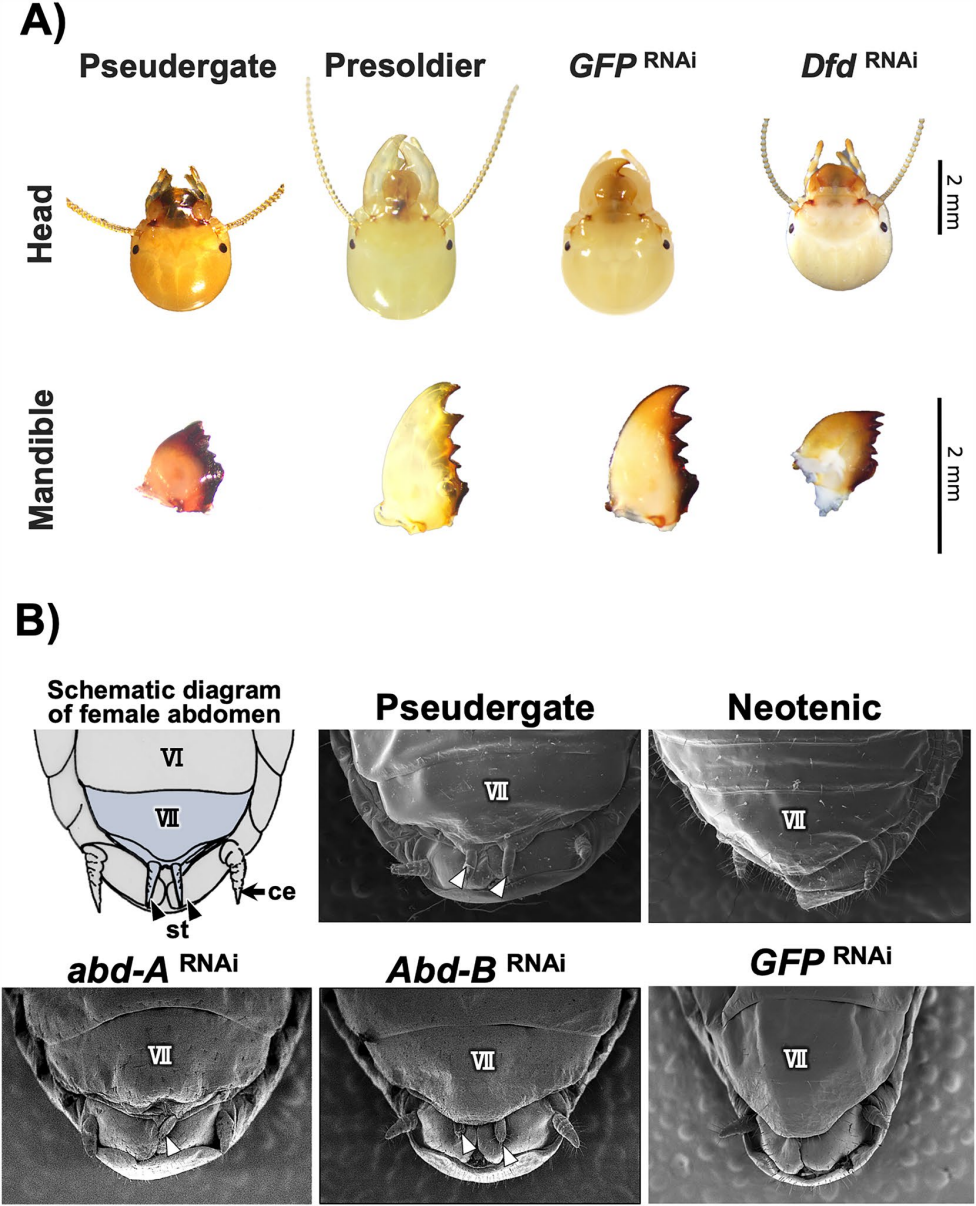
Functional analyses of effects of RNA interference of *Dfd* on soldier differentiation, and of *abd-A* and *Abd-B* on neotenic differentiation. **A** Head and mandible features in Pseudergate, Presoldier, *GFP* RNAi and *Dfd* RNAi-treated and molted individuals are shown. **B** Female Sternite morphologies were examined in each category: pseudergate, neotenic, *abd-A* RNAi-, *Abd-B* RNAi- *GFP* RNAi-treated individuals after molt. As indicated in schematic diagram “VII” indicates the seventh sternite, arrowhead denotes the styli (“st”) and arrow shows the cerci (“ce”)

To elucidate the functions of Hox genes specifically upregulated during three types of caste differentiation, RNAi was performed (Fig. 4). Morphological examinations focusing on molted individuals after *Dfd* RNAi showed smaller mandibles than those of *GFP* control individuals (Fig. 4A, Additional file 1: Fig S1). Furthermore, both *abd-A* and *Abd-B* RNAi resulted in molted individuals that possessed abnormal sternal morphologies (Fig. 4B). Both *abd-A* and *Abd-B* RNAi resulted in narrower seventh sternites and a pair of styli, which were characteristics of normal female pseudergates (Fig. 4B). The frequency of individuals with styli was significantly higher with Abd-B RNAi treatment than GFP RNAi (Fisher’s exact test, *abd-A* RNAi, *p* < 0.05; *Abd-B* RNAi, *p* < 0.05). Thus, *Dfd* RNAi disrupted soldier-specific mandibular morphogenesis and *Abd-B* RNAi disrupted neotenic-specific abdominal morphogenesis. Unfortunately, microinjection in nymphs led to regressive molt or death, so functional analyses were not carried out on the alate differentiation.

## Discussion

**Fig. 5:**
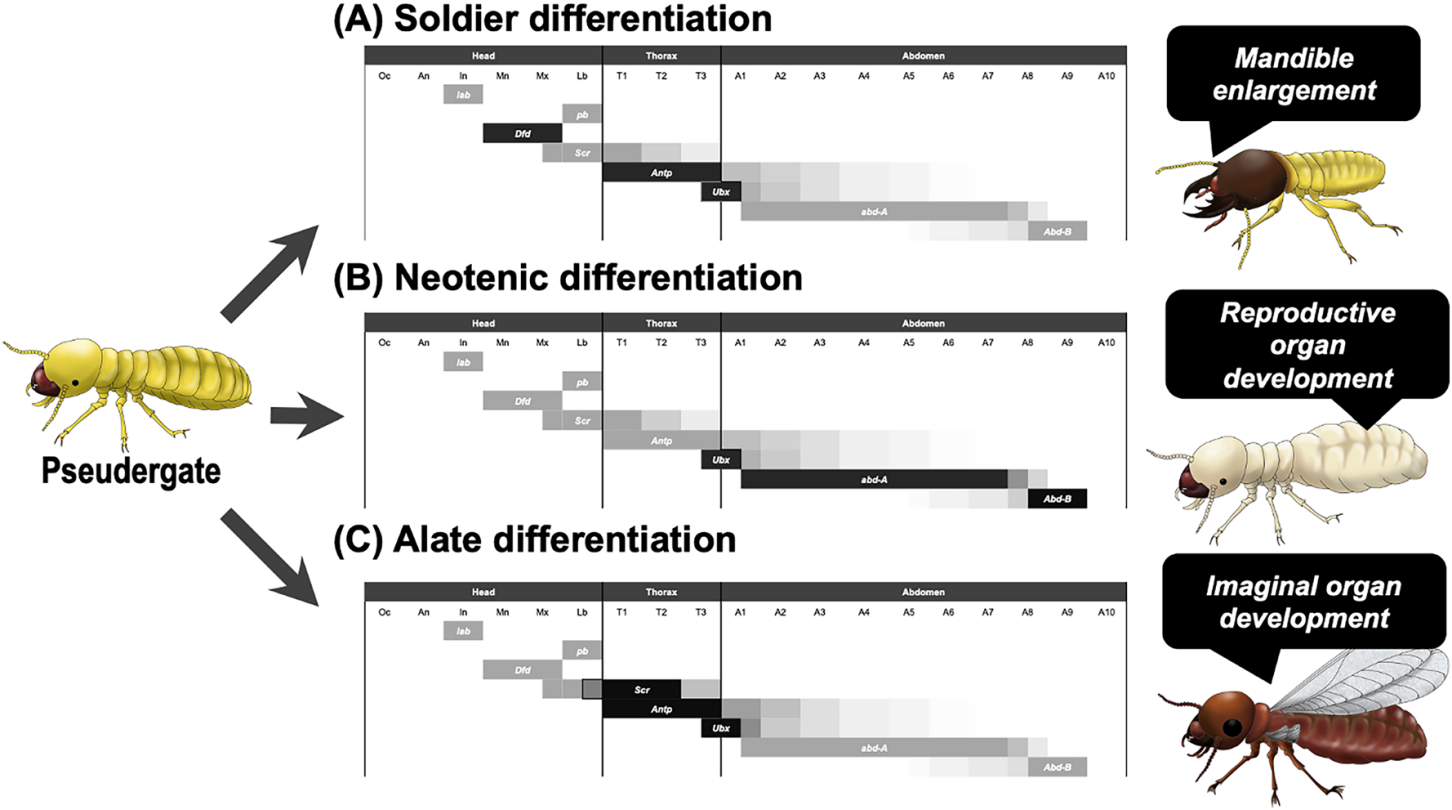
Schematic diagrams of the expression changes of Hox genes during the processes of caste differentiation. Based on the results in this study, together with previous studies (36; 38; 39), the Hox-gene repertoires expressed in each segment of the body are presented in the diagrams. The genes shown in darker colors are those that are upregulated during each caste differentiation process. The expression of *Dfd*, *Antp* and *Ubx* is upregulated during soldier differentiation (**A**), *Ubx*, *abd-A* and *abd-B* during neotenic differentiation (**B**), and *Scr*, *Antp* and *Ubx* during alate differentiation (**C**), controlling morphogenesis specific to each caste

Although it is generally known, in bilaterians, that the *Hox* genes play important roles for determining the identity of body parts along the anterior–posterior axis especially during embryogenesis [29–31, 34], our data showed that expressions of *Hox* genes were also maintained during post-embryonic processes and regulated in caste- and body part-specific manners (Figs. 2, 5). These results suggest that, for caste-specific morphogenetic processes, the expression of *Hox* genes would be required even during post-embryonic development.

In the soldier differentiation, only a few out of the eight Hox genes, i.e., *Dfd, Antp* and *Ubx*, were upregulated before the presoldier molt (Fig. 2A). Comparisons of expression patterns among body parts showed that only *Dfd* was specifically expressed in the head part, which was drastically modified during the soldier differentiation (Fig. 3). Furthermore, RNAi knockdown of *Dfd* strongly inhibited mandibular elongation in the caste differentiation process (Fig. 4). This finding, together with a previous study showing that *Dfd* was highly expressed specifically in mandibles before the presoldier molt [19, 35], suggests that the upregulation of *Dfd* contributes to mandibular elongation during soldier differentiation.

In contrast to some anterior *Hox* genes that were upregulated during soldier differentiation in termites, the bithorax complex genes (*Ubx, abd-A, Abd-B*) providing the posterior segmental identity were highly expressed before neotenic differentiation (Fig. 2B). Among those, *abd-A* and *Abd-B* were specifically expressed in the abdomen, and functional assays of these genes disrupted sternal morphogenesis during neotenic differentiation (Figs. 3B, 4B). Especially, RNAi-treated females in which *Abd-B* was knocked down possessed narrower seventh sternites and a pair of styli, which were characteristics of normal female pseudergates (Fig. 4B). This result indicates that upregulation of *Abd-B* might be responsible for structural modifications of posterior parts during neotenic differentiation. Consistently, it was also reported that, in a milkweed bug, *Oncopeltus fasciatus,* expression patterns of *Ubx, abd-A* and *Abd-B* were nested and overlapped along the anterior–posterior axis, and *abd-A* and *Abd-B* regulate normal development of posterior abdominal parts and genital structure development [36, 37].

In addition, expressions of three Hox genes, *Scr*, *Antp* and *Ubx*, were upregulated during alate differentiation (Fig. 2C). Among these genes, *Scr* and *Antp* are involved in determining thoracic identity during insect development [30, 38–42]. Our results also showed these genes were specifically expressed in the thorax during alate differentiation (Fig. 3C). Since wing and flight muscle development occurs in the thorax during alate differentiation [15], *Scr* and *Antp* were suggested to be involved in the morphological modifications of the thorax during alate differentiation (Fig. 3C). Unfortunately, functional analysis by RNAi was not possible because nymphs underwent regressive molt in response to the control RNAi procedure, probably due to the mechanical stimulus, i.e., injection (data not shown). The developmental stage of alate in termites is known to be homologous to the imaginal stage in ancestral solitary insects [27, 43]. The results in this study, in which Hox gene expressions changed during alate differentiation, together with those of previous studies [30, 38–42], suggest that expressions of Hox genes are maintained even after embryogenesis in insects. It is likely that Hox genes provide spatial information during post-embryonic development, inducing appropriate morphogenesis during large-scale morphological changes such as imaginal molt [37–39]. These functions of Hox genes during post-embryonic development may be “preadaptations” that enable diverse morphological modifications in social insects (i.e., caste polyphenism).

As shown above, upregulation of specific *Hox* genes is necessary for the caste-specific morphogenesis during caste differentiation processes (Fig. 5). It is known that, generally in embryogenesis, the expressions of Hox genes are regulated downstream of multiple inputs such as gap genes, segment polarity genes, pair-rule genes, etc., starting from maternal factors deposited in oocytes [29, 30]. In contrast, in post-embryonic processes, little is known about the regulatory mechanisms upstream of the Hox expressions. Although it is possible that the whole gene expression cascade occurring during embryogenesis could take place repeatedly in post-embryonic development, the results of this study, together with previous studies [19, 31, 35, 36, 38], suggest that the Hox expressions providing positional information are continuously maintained even during post-embryonic development. It was previously pointed out that the maintenance of Hox-gene expressions even during post-embryonic development can provide evolutionary potential to establish new developmental modules [44]. Considering that caste differentiation is regulated by endocrine factors such as juvenile hormone (JH) and ecdysone, the upregulation of certain Hox genes in post-embryonic processes is also controlled downstream of such endocrine systems [19, 21–23, 25–27]. Actually, it was shown that the expression of *Dfd* was regulated by the JH receptor gene *Met* in soldier differentiation [19]. Future studies will be required to unravel the cross-talks between endocrine downstream factors and *Hox* genes.

## Methods

### Insects

Colonies of *H*. *sjostedti* Holmgren (family Hodotermopsidae, previously family Archotermopsidae. [45] inhabiting rotten wood were collected on Yakushima Island, Kagoshima Prefecture, Japan, in every May from 2018 to 2022. Colonies were maintained in plastic containers with nest logs at ~ 25 °C under constant darkness and were occasionally fed moistened pinewood. Caste categories were identified based on previous studies in the focal species [14, 46].

### Induction of caste differentiation

To induce individuals to undergo soldier differentiation, a JH analog, pyriproxyfen, was applied [32]. As previously described [e.g., 19, 47], 10 pseudergates (Pe) were placed in a Petri dish (ø 70 mm) lined with filter paper containing 10 µg pyriproxyfen (Sigma-Aldrich, St. Louis, MO) and moistened with distilled water. Petri dishes were monitored daily and maintained at 25 °C under constant darkness. The presoldier molt typically occurred 14 days after JHA application (Fig. 1B). During soldier differentiation, samples were obtained at the 1 week after JHA application (JHA1w), 2 weeks after JHA application (JHA2w), and presoldier induced by JHA (JHAPS) stages. For comparison, natural presoldiers (NPSs) were also included to evaluate artificial effects that might be induced by the JHA application.

According to previous works [20, 33, 48], female neotenic differentiation was induced by artificially manipulating the sex ratio of reproductives in experimental colonies (Fig. 1C). Briefly, 20 pseudergates (10 females and 10 males) and a male neotenic were placed in a polystyrene case (9.5 × 6 × 2 cm) with sufficient food (a mixture of wood sawdust and cellulose powder moistened with distilled water). Most of the isolated female pseudergates molt into neotenics at around 14 days, and therefore female pseudergates (Pe) were collected at 1 week after induction (1w), 2 weeks after induction (2w) and neotenic (Neo) stages.

In the focal species, since alates emerge in June under natural conditions, nymphs with various developmental degrees can be collected in May [15]. Therefore, based on a previous study [15], nymphs without swollen wing buds (Ny), nymphs with whitish abdomen and just after the beginning of gut-purge (NyGp), nymphs with swollen wing buds (NyS) and alates 1 week after the imaginal molt (Ala) were collected (Fig. 1D).

### Identification of Hox gene orthologs

To identify eight Hox genes (i.e., *lab, pb, Dfd, Scr, Antp, Ubx, abdA and AbdB*) in the focal termite species, orthologous sequences were searched based in the NCBI database and the transcriptome data from *H. sjostedti* (DDBJ Sequence Read Archive: DRA005483) [19]. Among them, Antennapedia complex genes (i.e., *lab, pb, Dfd, Scr* and *Antp*) were identified in previous works [19]. To identify Bithorax complex genes (i.e., *Ubx, abdA* and *AbdB*), protein sequences of the corresponding candidate genes in *Drosophila melanogaster* were used as queries for the tBLASTn searches against the transcriptome datasets in the focal species. Based on the obtained sequences, primers for real-time qPCR were designed using Primer Express software (ver. 3.0.0, Applied Biosystems, Foster City, CA, USA; Table 1).

**Table 1:** Primer sequence lists for real-time qPCR

Gene name (symbol)	Primer sequences (forward)	Primer sequences (reverse)
*labial (lab)*	5′-AAGTACCTGACACGAGCGAGAAG-3′	5′-TCTGTTTCATGCGACGATTCTG-3′
*proboscipedia (pb)*	5′-CAGGTGGCGGGCTCAAC-3′	5′-CAACAGCCAGCCCTCTATGG-3′
*Deformed (Dfd)*	5′-GCTGGAGTAGCGAATGGATCA-3′	5′-GGAACTCTTTCTCCAGCTCCAGTA-3′
*Sex comb reduced (Scr)*	5′-AAACGCCAATGGAGAAACGA-3′	5′-TCTGGTCAGGTAGCGGTTGAA-3′
*antennapedia (antp)*	5′-CCCCTGGATGAGGAGTCAGTT-3′	5′-GTAGCGGTTGAAGTGGAATTCC-3′
*Ultrabithorax (Ubx)*	5′-AACGAACAGGAGAAGCAAGC-3′	5′-TTGTTGTTGCTGCGCTACTG-3′
*abdominal-A (abd-A)*	5′-GAGCCCTTTTGACAGAGTCG-3′	5′-GTGCATGGGCTATCTCGATT-3′
*Abdominal-B (Abd-B)*	5′-TCCTGTTCAACGCTTACGTG-3′	5′-TCTGGTTGTTAGCGTTGCTG-3′

### RNA extraction and quantitative polymerase chain reaction

To quantify expression levels during each caste differentiation, total RNAs were extracted from whole-bodies of individuals along the time course from each of the three individuals (Fig. 1B–D). To compare expression levels among body parts, total RNAs were also extracted from different body parts, including head, thorax and abdomen dissected from three individuals at 1 week after the induction of soldier differentiation, and at 2 weeks after the induction of neotenic differentiation. Total RNA was extracted using RNAiso Plus (Takara Bio, Shiga, Japan) according to the manufacturer’s protocol. After the extraction, samples were treated with DNase I (Thermo Fisher Scientific, Waltham, MA, USA).

For each sample, total RNA was reverse-transcribed with a High-Capacity cDNA Reverse Transcription Kit according to the manufacturer’s instructions (Applied Biosystems, Foster City, CA, USA). Quantifications of the relative expression levels were performed using Fast SYBR Green Master Mix and the sequence detection system ABI PRISM 7500 (Applied Biosystems, Foster City, CA, USA). For the reference gene selection, the suitability of different candidate reference genes (18S rRNA [18S], Glyceraldehyde-3-phosphate dehydrogenase [GAPDH], elongation factor 1 alpha [Ef1a], and ribosomal protein 49 [RP49]) were compared using the software geNorm [49] and Normfinder [50] and RP49 was selected as the most appropriate reference gene. Data acquisition and analyses were performed with ABI Prism 7500 software ver. 2.0.4 (Applied Biosystems, Foster City, CA, USA), with the relative standard curve method. For statistical analyses to detect significant differences in expression levels, respectively, Tukey’s multiple comparisons test (*p* < 0.05) was performed after one-way ANOVA (*p* < 0.05), using R 3.5.0 (https://www.r-project.org).

### RNA interference

To evaluate gene function, *Dfd* was knocked down by RNAi during the soldier differentiation and *abdA* and *AbdB* were knocked down during the neotenic differentiation process according to previous studies assessing caste differentiation in the focal species [19, 51]. Annealed siRNAs were produced by Japan Bio Services Co. Ltd. (Saitama, Japan). The siRNA sequences were as follows: *Dfd* (sense, 5′-GUA GUU AUG GGA AUU AUU ATT-3’′; antisense, 5′-UAA UAA UUC CCA UAA CUA CCA-3′), *abdA* (sense, 5′- CAU ACA CAG AGG AAG AAU-3′; antisense, 5′- AAU UCU UCC UCU GUG UAU-3′), and *AbdB* (sense, 5′-GUU GUA GUU GUA CUC CAG CTT-3′; antisense, 5′-GCU GGA GUA CAA CUA CAA CTT-3′). The siRNA was diluted with nuclease-free water to a final concentration of 20 pmol/μL. A volume of 1 μl siRNA (Dfd, *n* = 15; abdA, *n* = 15; AbdB, *n* = 15; and GFP, *n* = 15) was injected into termites. Transcript levels of target genes were quantified by real-time qPCR at 3 days after RNAi, in the head after induction of soldier differentiation and in the abdomen after induction of neotenic differentiation (Additional file 1: Fig S1). In the qPCR analyses, biological triplicates were quantified and subjected to Welch's t-test, and *p* < 0.05 was taken to indicate statistical significance. Only individuals that molted within 3 weeks were used for the morphological evaluation. Phenotypes of RNAi individuals were evaluated by sternal structure according to a previous study [20].

## Supplementary Information

**Additional file 1: Fig S1.** Effects of RNAi were evaluated by real-time qPCR. Asterisks above the bars denote significant differences from the GFP-RNAi control (Welch’s *t*-test, p < 0.05). The expression level of each gene was reduced by RNAi of the respective gene. Mandible length is compared in the right graph. Asterisks above the bars indicate significant differences compared to the GFP-RNAi control (Welch’s t-test, p < 0.05)

## Data Availability

The datasets used and/or analyzed during the current study are available from the corresponding author on reasonable request.
