# Supplementary figures and images for: Upregulation of Hox genes leading to caste-specific morphogenesis in a termite
Source: EvoDevo. 2023 Jul 27;14:12. doi: 10.1186/s13227-023-00216-w (PMC10375622; doi:10.1186/s13227-023-00216-w)

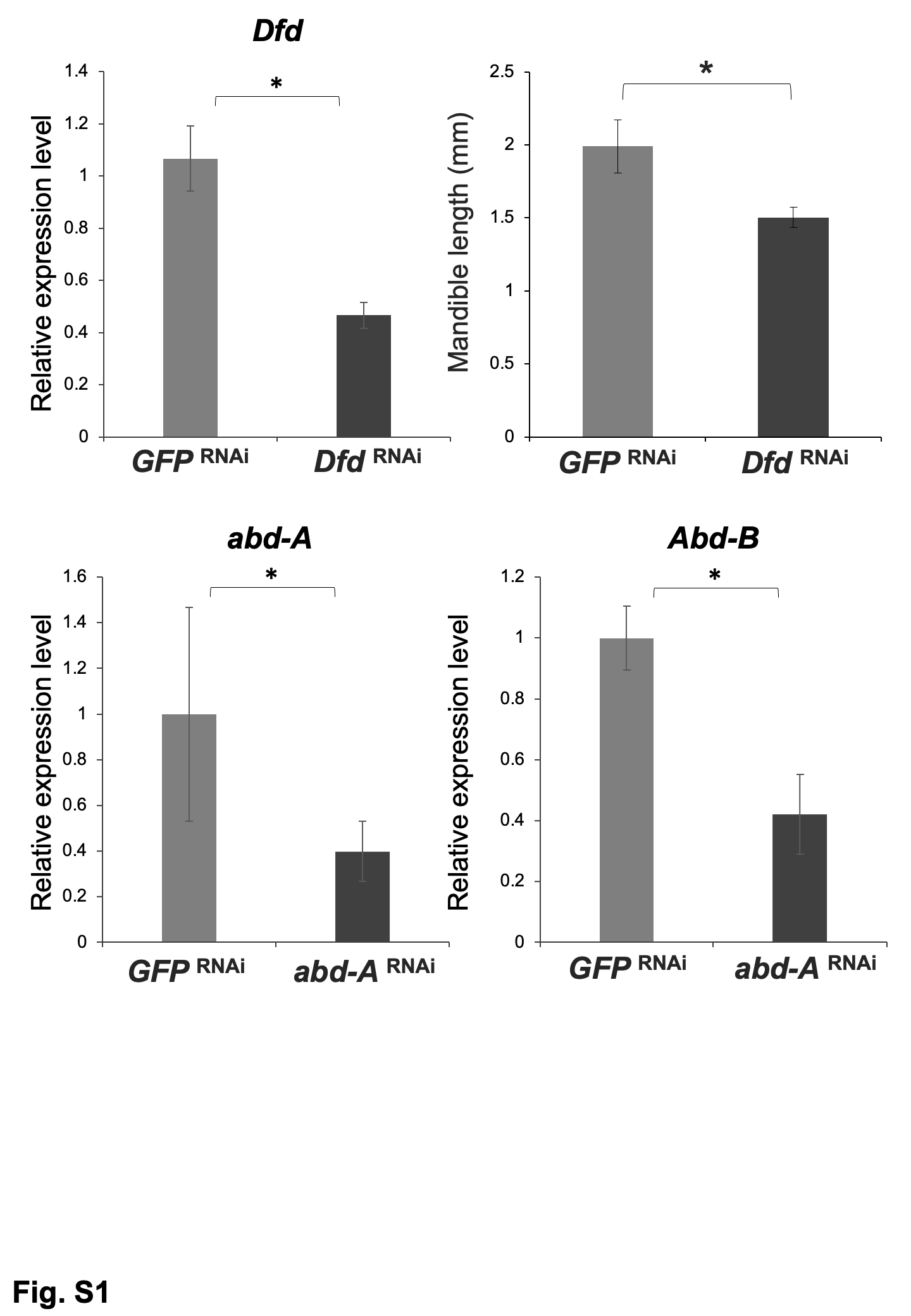

Supplement: Supplementary file 1 — Additional file 1: Fig S1. Effects of RNAi were evaluated by real-time qPCR. Asterisks above the bars denote significant differences from the GFP-RNAi control (Welch’s t-test, p < 0.05). The expression level of each gene was reduced by RNAi of the respective gene. Mandible length is compared in the right graph. Asterisks above the bars indicate significant differences compared to the GFP-RNAi control (Welch’s t-test, p < 0.05) [file 13227_2023_216_MOESM1_ESM.tiff]
